# Patience and Patients

**Published:** 1863-04

**Authors:** 


### Editorial.

---

### PATIENCE AND PATIENTS.

"FAITH worketh patience, patience experience, and experience hope." And this is as true in dental surgery as in religion. The man who has faith in his calling, faith in himself, faith in his fellow-men, is always a patient man. Having faith in his profession, he can calmly hear its utility called in question. Knowing that his motives are unselfish, he can afford to hear them impunged. Believing in his fellow-men, he will not condemn the race on account of the whims of individuals. Being a patient man, he is ever in proper condition to compare, reflect, and judge. His *experience* is practical and useful. He goes on from point to point, in the practical application of sound principles, and thus, making the attainments of each day surpass those of its predecessors, he learns to be *hopeful.* He *desires* to advance his calling, and to bless his race; and he *expects* to obtain the fulfillment of his desires. A man of this type is fit to practice a profession. Such a man will be a blessing to himself, and a benefit to mankind. "And in old age, when others fade," wrinkled though he may be, not a furrow will be found in his face, turned by the ploughshare of fretfulness or frowns.

Is such a man practical? None so much so. When a patient is ignorant of what he really needs, this man remembers that not long ago he was equally ignorant on the same subject. When the patient is unreasonable, he reflects that all are unreasonable about what they do not understand, and so he patiently enlightens him. If the patient is penurious, he pities him, and explains to him that the proposed operation is far more than an equivalent for the proposed price. If the patient insists that his neighbor's prices are lower than his, he good-naturedly tells him that his neighbor is a conscientious man, and knows that his work is not worth as much as that of other people. If the patient is self-willed, obstinate, and will not yield to his judgment, he patiently bows him out.

But after all, the dentist has not much to try his patience. He has almost no care, no responsibility, no trials, when contrasted with the physician. On this point we are able to speak from experience. Hence, we make but little allowance for the dentist who frets about his calling, who becomes angry at his patients, who is unwilling to take a little time to explain what, to him, may seem a small matter, while to the patient it may seem decidedly otherwise. All men—and especially all women—have whims. When their gratification is harmless, the patient man—the true gentleman—is willing to gratify them. He expects his patients to humor his whims, and it is his duty to reciprocate. A man has as much right to his eccentricities as he has to the corns on his toes. It is well enough to remove a corn, but not by cutting off the toe. So the dentist may, by gentle means, if practicable, remove a whim of his patient, but never in such a way as to wound his feelings.

The patient, hopeful man, is far more likely to become a good operator than the one who is fretful and despondent; and he is far more likely to be appreciated by the public. W.

